# Biocontrol potential of endophytic fungi against phytopathogenic nematodes on potato (*Solanum tuberosum* L.)

**DOI:** 10.1038/s41598-024-64056-x

**Published:** 2024-07-05

**Authors:** Rehab Y. Ghareeb, Mariusz Jaremko, Nader R. Abdelsalam, Mohamed M. A. Abdelhamid, Eman EL-Argawy, Mahmoud H. Ghozlan

**Affiliations:** 1 Plant Protection and Biomolecular Diagnosis Department, Arid Lands Cultivation Research Institute (ALCRI), City of Scientific Research and Technological Applications (SRTA-City), Alexandria, Egypt; 2 Division of Biological and Environmental Sciences and Engineering (BESE), King Abdullah University of Science and Technology (KAUST), 23955 Thuwal, Saudi Arabia; 3 Agricultural Botany Department, Faculty of Agriculture (Saba Basha), Alexandria University, Alexandria, 21531 Egypt; 4 Department of Plant Pathology, Faculty of Agriculture, Damanhour University, Damanhour, Egypt

**Keywords:** *Aspergillus flavus*, Efficacy, Egg hatching, Root knot-nematode, Potato, Defense system response, Enzymes, GC–MS, Secondary metabolites, Biological techniques, Plant sciences

## Abstract

Root-knot nematodes (RKNs) are a vital pest that causes significant yield losses and economic damage to potato plants. The use of chemical pesticides to control these nematodes has led to environmental concerns and the development of resistance in the nematode populations. Endophytic fungi offer an eco-friendly alternative to control these pests and produce secondary metabolites that have nematicidal activity against RKNs. The objective of this study is to assess the efficacy of *Aspergillus flavus* (ON146363), an entophyte fungus isolated from *Trigonella foenum-graecum* seeds, against *Meloidogyne incognita* in filtered culture broth using GC–MS analysis. Among them, various nematicidal secondary metabolites were produced: Gadoleic acid, Oleic acid di-ethanolamide, Oleic acid, and Palmitic acid. In addition, biochemical compounds such as Gallic acid, Catechin, Protocatechuic acid, Esculatin, Vanillic acid, Pyrocatechol, Coumarine, Cinnamic acid, 4, 3-indol butyl acetic acid and Naphthyl acetic acid by HPLC. The fungus was identified through morphological and molecular analysis, including ITS 1–4 regions of ribosomal DNA. In vitro experiments showed that culture filtrate of *A. flavus* had a variable effect on reducing the number of egg hatchings and larval mortality, with higher concentrations showing greater efficacy than Abamectin. The fungus inhibited the development and multiplication *of M. incognita* in potato plants, reducing the number of galls and eggs by 90% and 89%, respectively. *A. flavus* increased the activity of defense-related enzymes Chitinas, Catalyse, and Peroxidase after 15, 45, and 60 days. Leaching of the concentrated culture significantly reduced the second juveniles’ stage to 97% /250 g soil and decreased the penetration of nematodes into the roots. *A. flavus* cultural filtrates via soil spraying improved seedling growth and reduced nematode propagation, resulting in systemic resistance to nematode infection. Therefore, *A. flavus* can be an effective biological control agent for root-knot nematodes in potato plants. This approach provides a sustainable solution for farmers and minimizes the environmental impact.

## Introduction

Potato (*Solanum tuberosum* L.) is considered a vital root crop, the fourth staple food, and an economically critical food crop after maize, rice, and wheat^1,2^. About 80% of the world's potato production is cultivated in several regions, including Europe, America, and Asia^3^. Potato is grown in an area of 20 million hectares and produces nearly 400 million tons annually^4,5^. In Egypt, 6.2 million tons of potatoes are from the cultivated area of 211 thousand hectares, with an average yield of 24.100 t ha^-1^ and a self-sufficiency rate of 111.4%^6^. Currently, there is an increasing need for food supply and food security. Unsustainable crop production approaches with monocultures, intensive planting, and expansion of crops into newly opened areas have enhanced problems related to new pests and diseases^7,8^; one of these essential crops is potato which infected by different pests and pathogens causing high yield losses such as *Globodera* spp., *Meloidogyne* spp., *Ditylenchus* spp. and *Pratylenchus* spp which are recognized as the main parasites infected potato^9,10^. *Meloidogyne* spp or nematodes are the most critical factors in reducing quantitative and qualitative yields in potatoes^11,12^. Nematodes are a restricting factor for potato production, reducing yields and chemical changes in potato tubers^5,10^. Nematodes caused an average yield loss of up to 12% in potatoes^13–15^. Moreover, many other species of infected potatoes, such as *Belonolaimus*, *Longidorus*, *Xiphinema*, *Rotylenchulus* spp, and *Radopholus similis*, are considered of minor importance in potato production^16^.

Recently, biological control of plant pests has increased^17,18^; most biological control endophyte (BCE) agents are commonly found among crops and can suppress critical pathogens^19,20^. Fungal pathogens have a harmful effect on plant physiology, which increases host defense responses to pathogens^21^. Fungi produce bioactive molecules, such as small peptide effectors, enzymes, and secondary metabolites that assist colonization and contribute to both symbiotic and defense against pathogenic relationships^22^. *Trichoderma* species, *arbuscular mycorrhizas*, *ectomycorrhizas*, endophytes, and yeasts of specific pathogens are among the significant beneficial fungi with biocontrol capacity^23,24^. Microbial pesticide active ingredients are microorganisms such as fungi, bacteria, viruses, protozoans, or algae with a broad spectrum of activity. However, every organism is relatively specific to its target pest^25,26^.

Microorganisms and nematodes interact with plant host roots to obtain their nutrients^27,28^. This interactive relationship is not unidirectional but multi-directional, with all these organisms interacting with each other either singly or in combination^29,30^. The fungus culture activity includes nematicidal effects on adult nematodes, inhibition of egg hatching and juvenile development^31^. Culture filtrates of numerous soil-borne fungi exhibited nematode-toxic characteristics^32^, though most of the initial studies were pointed to in vitro tests^33,34^. There is comparatively little information on the toxic effects of fungal metabolites on the development and reproduction of root-knot nematodes on plant growth^35^. Therefore, the objectives of the current study are to evaluate the efficacy of *Aspergillus flavus*, an entophyte fungus isolated from *Trigonella foenum*-*graecum* seeds, against *Meloidogyne incognita*.

## Materials and methods

### Source of endophytic *fungi* (Phytopathogens used for antagonistic assays)

The seeds of *Trigonella foenum-graecum* were sterilized using a sequential immersion method, which involved 1 min in 75% ethanol, followed by 10 min in 65% commercial Chlorox (final concentration of 3.25% aqueous sodium hypochlorite), and finally, a 30-s immersion in 75% ethanol^36–38^. The sterilized seeds were planted on potato dextrose agar (PDA) medium supplemented with cidocetine (500 mg/L). As well, the endophytic fungus was sub-cultured in demand to gain pure cultures. Identification of the fungal strain was detected using morphology of cultures or hyphae, the characteristics of the spores and reproductive structures if the characteristic, were visible. The isolated pure fungus was cultured on a potato dextrose agar plate and inoculated at 26 ± 2 °C in complete darkness After 5 to 7 day, about 5 mm diameter mycelia disc of fungus was inoculated into 250 mL of PDB in a 500 mL Erlenmeyer flask and incubated at 26 ± 2 °C for one week. The culture filtrate of *A. flavus* was prepared by centrifuging at 5000 rpm/15 min^39^. 10 ml of the supernatant was filtered by sterile 0.22 μm filter (Whatman, Clifon, NJ, USA) for the In vitro and In vivo experiment.

### Molecular characterization

#### Preparation of DNA and ITS-PCR conditions

*A. flavus* mycelium was harvested from a potato dextrose liquid medium and was ground in liquid nitrogen using a pestle mortar to extract DNA^40^. Amplifications were carried out in PCR reactions in a total volume of 20 μl, containing 2 µl genomic DNA; 12.5 master mix PCR; and 8 pmol of specific primer ITS1 (forward): 5′-TCCGTAGGTGAACCTGCGG-3′ and ITS4 primer (Revers): 5′- TCCTCCGCTTATTGATATGC-3′ was done^41^.

PCR amplification was carried out according to the following temperature: 55 s at 94 °C as an initial step, 40 cycles of 60 s at 94 °C, 90 s at 52 °C, and 5 min at 72 °C, and a final step of 5 min at 72 °C. Electrophoresis of PCR-amplified products was performed in 1% agarose for 50 min at 80–100 Volt. PCR products were stained with 2 µl of Ethidium Bromide and visualized with 250 nm ultraviolet light. A Gel purification kit was used to amplify the purified DNA, and the purified DNA product was collected in a 2 ml Eppendorf plastic tube and stored at – 20 °C for sequencing. The PCR products were forwarded to Macrogen sequencing service (Macrogen Inc. Seoul, South Korea) to perform direct sequencing of both DNA strands.

### Identification of fungal isolation

The BLAST program available from NCBI was used to identify the isolate by aligning the forward and reverse DNA strand sequences^42^. The amplified ITS rDNA fragment sequence was compared with NCBI non-redundant nucleotide sequence database using BLAST. Isolate identification was based on his analysis of megaBLAST output, which identified highly similar sequences. The hits of ITS rDNA sequences were then used in a phylogenetic analysis to determine the evolutionary relationship among the isolates and hits based on their ITS rDNA sequence^43^.

### Phylogenetic analysis

The BLAST program was used to find similar sequences in GenBank using the sequence as a query. The most similar reference sequences were found and prepared for further phylogenetic analyses. These sequences were associated with the program CLUSTALX^44^, and the MEGA software was used to construct the relevant phylogenetic tree^45^. Ambiguous positions were removed, and gap positions were deleted manually. The neighbor-joining method was used to analyze the alignment data, with 1,000 iterations of random stepwise sequence addition performed for each query. Bootstrap analysis with 1,000 replications was used to estimate statistical support for the internal branches.

### Phytopathogenic nematodes cultures and antagonistic assay In vitro

*M. incognita* was maintained on the roots of susceptible tomato plants (cultivar Alisa) in sterilized soil at 25 ± 2^◦^C in a greenhouse. The nematode eggs were extracted from the tomato roots. Each day, second-stage juveniles (J2s) of *M. incognita* were taken from the eggs and kept at a temperature of 15˚C. This process provided a consistent and abundant source of *J2s* and egg masses for use in the laboratory and greenhouse conducted by^46,47^. Freshly hatched juveniles were utilized in experiments to detect juvenile mortality activity. The J2s of *M. incognita* were utilized to detect the nematicidal activities of fungal filtrate. To prepare the enrichment media broth of a fungal strain, potato dextrose broth (PDB) medium was used and shaken at 180 rpm for five days at a temperature of 28 ± 2 °C. After centrifugation at 5000 × g for 10 min and filtering with blotting paper. The supernatant (spore suspensions) was prepared, and their concentrations (100, 75, 50, 25, and 10%) were modified to 10^8^ spores ml^−1^ and then examined against J2s and eggs hatchability (EH) of RKN. A nematode suspension containing 50 J2s was added to each vial with 9 mL of the prepared fungal cell-free supernatant. In contrast, another vial contained only the J2s suspension mixed with PDB medium as a control. Each treatment had three vials and was incubated at 25 ± 2 °C for 72 h. The number of dead and alive J2s was observed using a stereoscopic microscope at different time intervals from exposure time. Dead nematodes were identified if they did not move when probed with a fine needle, while active ones moved or appeared as winding shapes. The nematodes in each treatment were then transferred to distilled water for another 48 h to determine whether the dead ones regained mobility. The mortality (%) of J2s activity was calculated using the formula^48^:

$$\text{Mortality }\left(\text{\%}\right)=\frac{\text{Mortality in treatment}-\text{ Mortality in control}}{\text{Mortality in control}}\times 100.$$

The concentrations (100, 75, 50, 25, and 10%) that showed the strongest nematicidal activity against J2s of *M. incognita* were selected for the greenhouse experiment, which was conducted three times by examining its effects on J2s mortality as described above.

### Identification of bioactive compounds

#### High-performance liquid chromatography (HPLC)

To prepare the HPLC analysis standard, rutin powder was dissolved in methanol at a concentration of 1 mg/ml in a volumetric flask. The obtaining stock solution was then filtered using a 0.45 μm filter and sonicated for 2 min at 150 °C in ice before being injected into the HPLC system. Chromatography was conducted using an LC 20AD liquid chromatography system with a Shimadzu SPD-M20A PDA detector and C18G 120 (250 × 4.60) mm 5 μm column. A sample volume of twenty μl was injected, and the mobile phase consisted of a mixture of acetonitrile and 0.3% formic acid (15:85 v/v). The flow rate was set at 0.4 ml/min, and detection was performed at a wavelength of 350 nm^49^.

#### GC–MS analysis of endophytic fungal culture filtrate

The chemical constituents of the culture filtrate of *A. flavus* were identified through GC–MS analysis using an Agilent 7693 series GC system (United States) equipped with an OV-5 capillary column and an Agilent 5975C network selective mass detector^50^. The endophytic fungal culture filtrate was prepared by soaking the dry fungal culture filtrate in the eluent three consecutive times. It was subjected to GC–MS analysis using a Perkin Elmer instrument. The mass spectrometer was operated in the electron impact (El) mode at 70 eV in the scan range of 60–600 m/z.

#### Samples extraction for HPLC

To extract samples for HPLC analysis, 1 gm of fungal pellet was mixed with 10 mL of 80% methanol containing 1% acetic acid. The mixture was homogenized and placed in a tightly capped test tube, which was then shaken for 48 h at 200 rpm. After centrifugation and filtration through a 0.45 μm filter, the final filtrate was analyzed for phenolic compounds. This extraction process was performed in triplicate independent samples. Standard compounds such as Gallic acid, Protocatechuic acid, Catechin, Esculatin, Vanillic acid, Pyrocatechol, Coumarin, Cinnamic acid, 4,3-indol butyl acetic acid and Naphthyl acetic acid were used to detect the presence of phenolic and flavonoid compounds in the methanol extract of the *A. flavus* cultural filtrate pellet. The HPLC instrument and its conditions were previously described in studies^51,52^.

### Efficacy of fungal culture filtrate against *M. incognita* on potato plants (greenhouse experiment)

A greenhouse experiment was conducted to determine the effect of *A. flavus* culture filtrate on controlling *M. incognita* infecting potato cv. Nicola. The potato seeds were prepared for planting by undergoing surface sterilization with 1.25% sodium hypochlorite for five minutes, followed by rinsing in sterile distilled water. The soil was autoclaved at 121 °C for two hours and aerated for a week before used to fill pots. The potato seeds sown in pots filled with a soil and sand mixture (1:1 v/v) and irrigated as needed. After one week, the top 3 cm of soil near the potato roots removed before inoculating the plants with two thousand freshly hatched juveniles of *M. incognita* in a juvenile suspension. Following transplantation and inoculation, the potato plants received treatments, including abamectin application and four concentrations of *A. flavus* culture filtrates. Growth parameters, such as root and shoot length, weight, and nematode parameters, including number of galls, egg masses per plant, and J2s per 250 cm^3^ of soil, assessed after 60 days of growth. Egg masses counted by examining three root samples under a stereoscope after shaking cut roots in NaClO solution. The experiment conducted with five replicates for each treatment and repeated twice at the City of Scientific Research and Technological Applications, Egypt (SRTA-City) greenhouse^53,54^.

### Enzyme activity assays

Enzymatic activities of Chitinase, Peroxidase, and Catalase were measured 15, 30, and 60 days after nematode inoculation according to the method outlined by^55^. Initially, twenty-five grams of chitin milled and then suspended in 250 ml of 85% phosphoric acid (H_3_PO_4_). This mixture stored at 4 °C for 24 h before the blended in 2 L of distilled water using a warning blender. The resulting suspension was centrifuged, and this washing process repeated twice. The final wash resulted in a colloidal chitin suspension that adjusted to pH 7.0 with (1 N) NaOH. This suspension separated by centrifugation, and the pelleted colloidal chitin stored at 4 °C. The determination of Chitinase activity was conducted using the^56^ method. Initially, 1 ml of 1% colloidal chitin in 0.05 M citrate–phosphate buffer (pH 6.6) was placed in test tubes, followed by 1 ml of enzyme extract, mixed by shaking. The exact process repeated using crude extract instead. The tubes incubated in a water bath at 37 °C for 60 min, cooled, and centrifuged before assaying. The amount of reducing sugar in 1 ml of the supernatant was determined using dinitrosalicylic acid^57^, with optical density measured at 540 nm. Chitinase activity was expressed as mM N-acetylglucosamine equivalent released per gram fresh weight tissue per hour. Peroxidase activity was determined spectrophotometrically by the methods of^58^, while, Catalase was according to^59^.

### Statistical analysis

The statistical analysis of the data involved the use of analysis of variance (ANOVA) and a revised LSD test to determine significant differences among the means at a *p-value* of ≤ 0.05. The SAS system^60^ was utilized for this purpose.

### Ethics approval and consent to participate

This article does not contain any studies with human or animal subjects. The current experimental research and field study, including collecting plant material and compliance with relevant institutional, national, and international guidelines and legislation, is used for research and development.

## Results

### Identification of the isolated endophytic fungus

**Figure 1:**
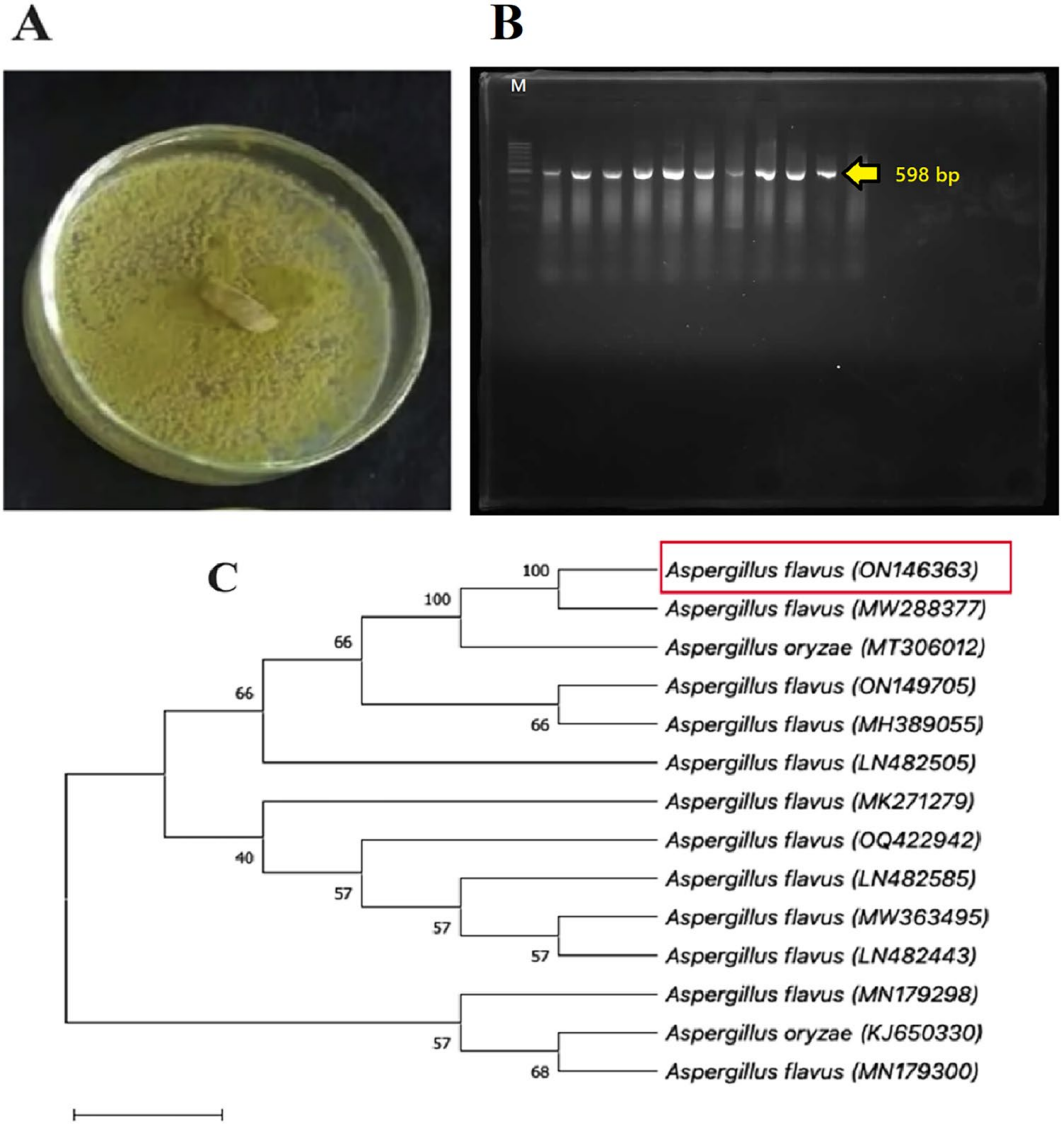
(**A, B**) Polymerase chain reaction (PCR) of *Aspergillus flavus* with ITS1-ITS4 (598 bp) and (**C**) Neighbor-joining tree of fungal endophyte *A. flavus* ON146363 based on ITS1-5.8S-ITS2 rDNA sequences. Confidence values above 50% obtained from a 500-replicate bootstrap analysis shown at the branch nodes. Bootstrap values from the neighbor-joining method were detected. *A. flavus* (LN482443) was used as the outgroup.

The isolation process led to the identification of endophytic fungal isolate from the genus Aspergillus, as confirmed by initial microscopic analysis (Fig. A). The isolated Aspergillus specimen was determined to be *Aspergillus* sp. The colonies of *A. flavus* exhibited rounded shapes, a velvety texture, a wet appearance, and a yellowish-green color with a light white border on PDA media (Fig. 1). In addition, the confirmation of the fungus isolate as *Aspergillus flavus* was determined through analysis of ITS sequence and culture morphology. Identification of the endophytic isolate fungal was identified by DNA sequences of the purified PCR product to confirm the genus of the microscopically identified *A. flavus* isolate, isolated from *T. foenum-graecum* seeds, the *Aspergillus* consensus ITS1-ITS4 PCR fragment was amplified (*Aspergillus flavus* ON146363). ITS sequencing region and identification of endophytic fungs were aligned, and the alignment sequence was subject to homology search by NCBI BLAST. Homology of the ITS region was used to identify the isolated endophyte as a species of *Aspergillus*. The current sequence showed 100% homology with sequences of the ITS regions of *Aspergillus* available in the Gene Bank database using the BLAST search program (https://blast.ncbi.nlm.nih.gov/Blast.cgi). To check the suitability of the isolated genomic DNA in downstream molecular biology applications, fungal strains were subjected to amplification using universal primers for the ITS region of 18 S rDNA^61^.

Figure 1A and B presented the gel electrophoresis and phylogenetic tree of the isolated endophytic fungi. The GenBank search for similar ITS region sequences revealed that strain ON146363 had higher sequence similarities with the species *A. flavus* than any other reference taxa. All ten sequences of reference taxa were acquired for constructing phylogeny. In the neighbor-joining (NJ) tree (Fig. 1C), strain ON146363 and the other reference taxa, *A. flavus* (MW288377), formed a clade with 99% bootstrap reinforcement. In this clade, the results of similarity comparative with the ITS1- 5.8S-ITS2 part sequence show that strain ON146363 had the maximum nucleotide similarities with *Aspergillus flavus*. This indicates that the morphological and molecular identification results could be assured that ON146363 is *A. flavus*.

### In vitro antagonistic activity against J2s mortality of *M. incognita*

**Figure 2:**
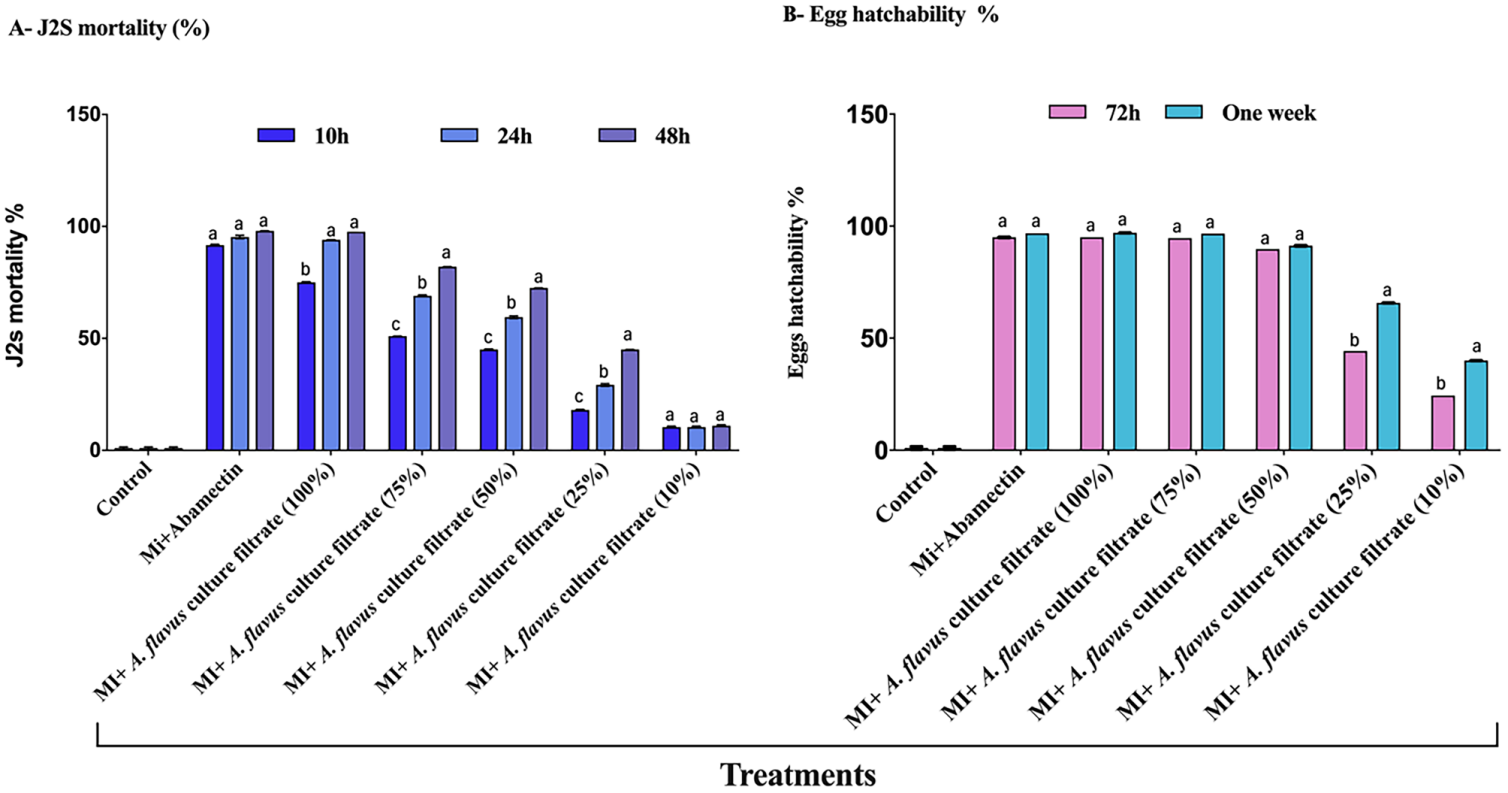
Effect of fungal culture filtrate on (**A**) larvae mortality and reduction percentages (R%) of the root-knot nematode and on (**B)** hatchability of *M. incognita* eggs (R%), under laboratory conditions after 12, 24, 48, 72 h and one week of exposure time. Bar charts are generated using data from five biological replications with two technical replicates per replication. Each bar represents the mean, and the error bar indicates the standard deviation (± SE). This figure was drawn with GraphPad Prism 8 (9.4.1, (458) Serial number: GPS-2567891- 8A130A8A228). The significant of the changes were assessed using SAS one-way ANOVA test. Means with different letters are significantly different (P ≦ 0.05).

The mortality rates of second-stage juvenile nematode odes were directly proportional to the concentration of culture filtrate, as shown in Fig. 2A. The appearance of J2s was not uniform when exposed to fungal culture filtrates, and all treatments showed a significant (P ≤ 0.05) increase in J2s mortality percentage after 12, 24, and 48 h compared to the control (DH2O). However, the response varied at different concentrations of the fungus culture filtrate treatments. The highest mortality rate for J2s was observed at the maximum concentration (100%), while the lowest concentration resulted in lower mortality rates. In most concentrations, the hatching of nematode eggs was significantly reduced compared to the control with DH2O. Culture filtrates from *A. flavus* caused 100% juvenile mortality after 48 h of exposure at a concentration of 100%. The differences in values between each concentration level were significant, with a stepwise increase in J2s mortality percentages with increasing concentrations and exposure time. The most aggressive concentration appeared to be *A. flavus* culture filtrate with a concentration of 100%, which caused 75%, 94%, and 97.7% juvenile mortality of J2s larvae in vitro after exposure times of 12, 24, and 48 h, respectively.

### In vitro effect on egg hatching of *M. incognita*

The results of the egg hatchability bioassay (Fig. 2B) demonstrate that the culture filtrates of *A. flavus* had a strong inhibitory effect on the hatching of *M. incognita* egg masses. The percentage decrease in hatchability ranged up to 95.1% and 97% after 72 h and one week of exposure, respectively. The various concentrations of the culture filtrate showed significant differences compared to the water control, with only the 10% dilution showing no significant difference. The data revealed that *A. flavus* culture filtrate was detrimental to egg hatching, with the lowest rate of hatchability observed at all concentrations tested. Moreover, the efficacy of the fungus culture filtrate in causing juvenile mortality was highest at 100%, followed by 75%, 50%, and 25% at the same time.

### Efficacies of fungus culture filtrate against *M. incognita* on potato plants (greenhouse experiment)

**Figure 3:**
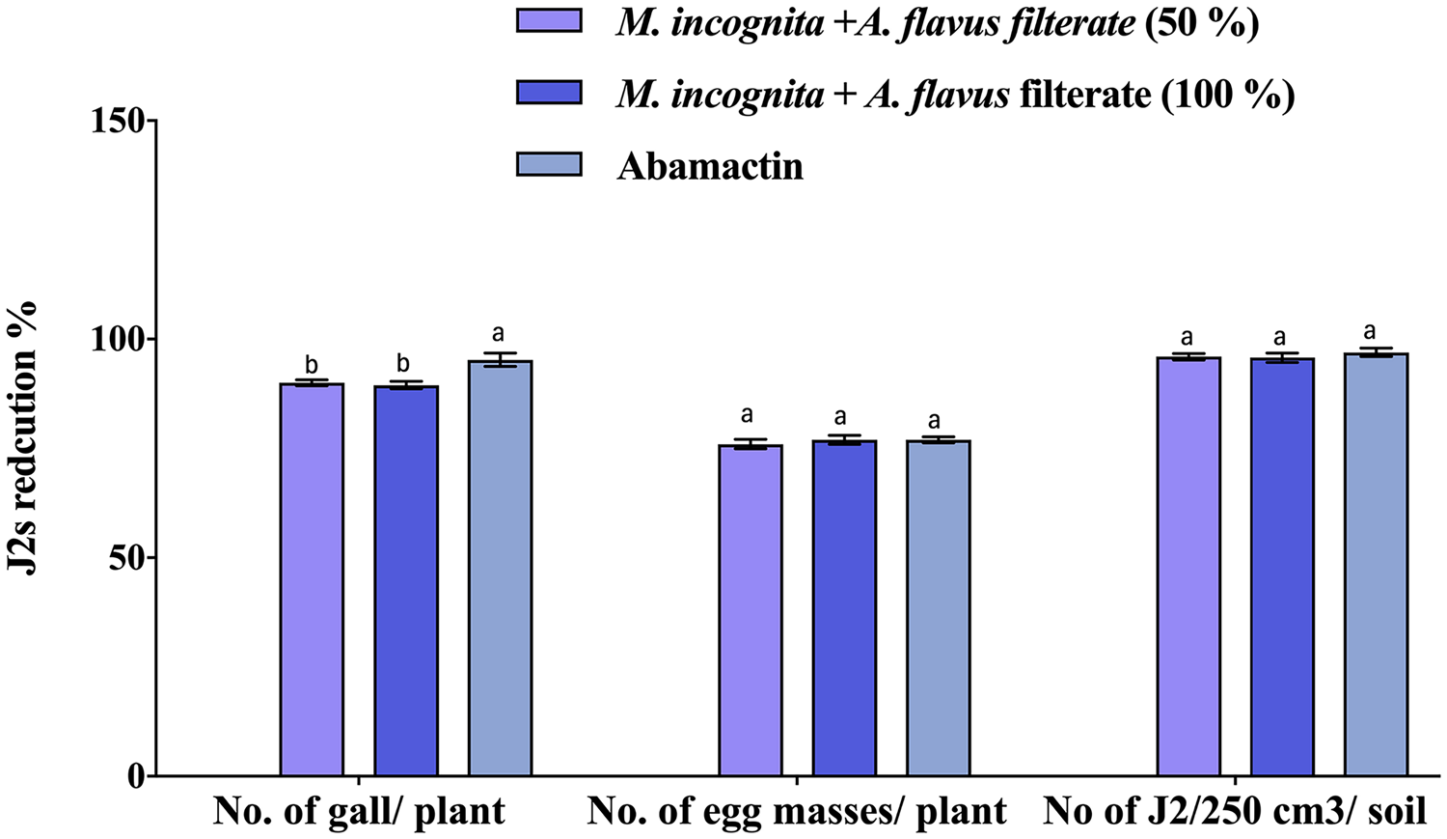
The impact of *A*. *flavus* culture filtrate on *Meloidogyne incognita* galls, egg masses, and J2s in 250 g soil of potato plants was examined under greenhouse conditions. Bar charts are generated using data from three biological replications with two technical replicates per replication. Each bar represents the mean, and the error bar indicates the standard deviation (± SE). This figure was drawn with GraphPad Prism 8. The significant of the changes were assessed using SAS one-way ANOVA test. Means with different letters are significantly different (P ≦ 0.05).

The effectiveness of fungus culture filtrate in controlling *M. incognita* on potato plants was evaluated through a greenhouse experiment based on the *Invitro* nematicidal efficiency, and the disease control activity of fungal culture filtrate was assessed with potato root-knot nematode diseases in pot experiments. Also, the tested fungal culture filtrate 100% (s) concentration was potent and influential in suppressing the formation of the galls, the number of egg masses/ plant, and the number of J2s/250 cm3 of soil with potato roots by 90, 89.5 and 95.3%, respectively, compared with the Abamectin, (Fig. 3). The influence of fungal filtering and the commercial nematicide (Abamectin) on growth parameters of nematode-infected potatoes under greenhouse conditions were illustrated in (Table 1).

**Table 1:** Effect of *A. flavus* culture filtrate against the root-knot nematode, *M. incognita* galls, egg masses, and J2s /250 g soil of potato plants under greenhouse conditions.

Treatment parameters	Length/cm				Fresh weight/g			Dry weight/g			
	Shoot	*I*	Root	*I*	Shoot	Root	*I*	Shoot	*C*	Root	*C*
Control (Healthy)	35.4^c^ ± 25		25.8^ cd^ ± 0.08	-	39.5^c^ ± 0.01	9.2^d^ ± 0.4	-	3.92^ cd^ ± 0.004	-	2.22^bc^ ± 0.53	-
*M incognita* + *A. flavus* culture filtrate (100%)	48.8^a^ ± 41	37.85	31.7^a^ ± 0.07	22.86	48.2^a^ ± 0.02	15.8^a^ ± 0.38	31.41	7.35^a^ ± 0.032	87.5	2.8^a^ ± 0.44	26
*M. incognita* + *A. flavus* culture filtrate (50%)	42.8^b^ ± 408	20.62	29.4^b^ ± 0.1	13.95	43.1^b^ ± 0.04	13.3^bc^ ± 0.408	15.81	4.82^c^ ± 0.01	23.5	2.42^bc^ ± 0.004	9
*Abamectin*	47^ab^ ± 41	32.7	30.9^a^ ± 0.04	19.7	43.6^b^ ± 0.014	13^bc^ ± 0.2	16.22	5.8^b^ ± 0.02	48.5	2.7^a^ ± 0.022	21.6

*I: increase percentage; control infected control; means followed by the same letter are not significantly different by Duncan’s multiple range tests (P ≤ 0.05).

**Figure 4:**
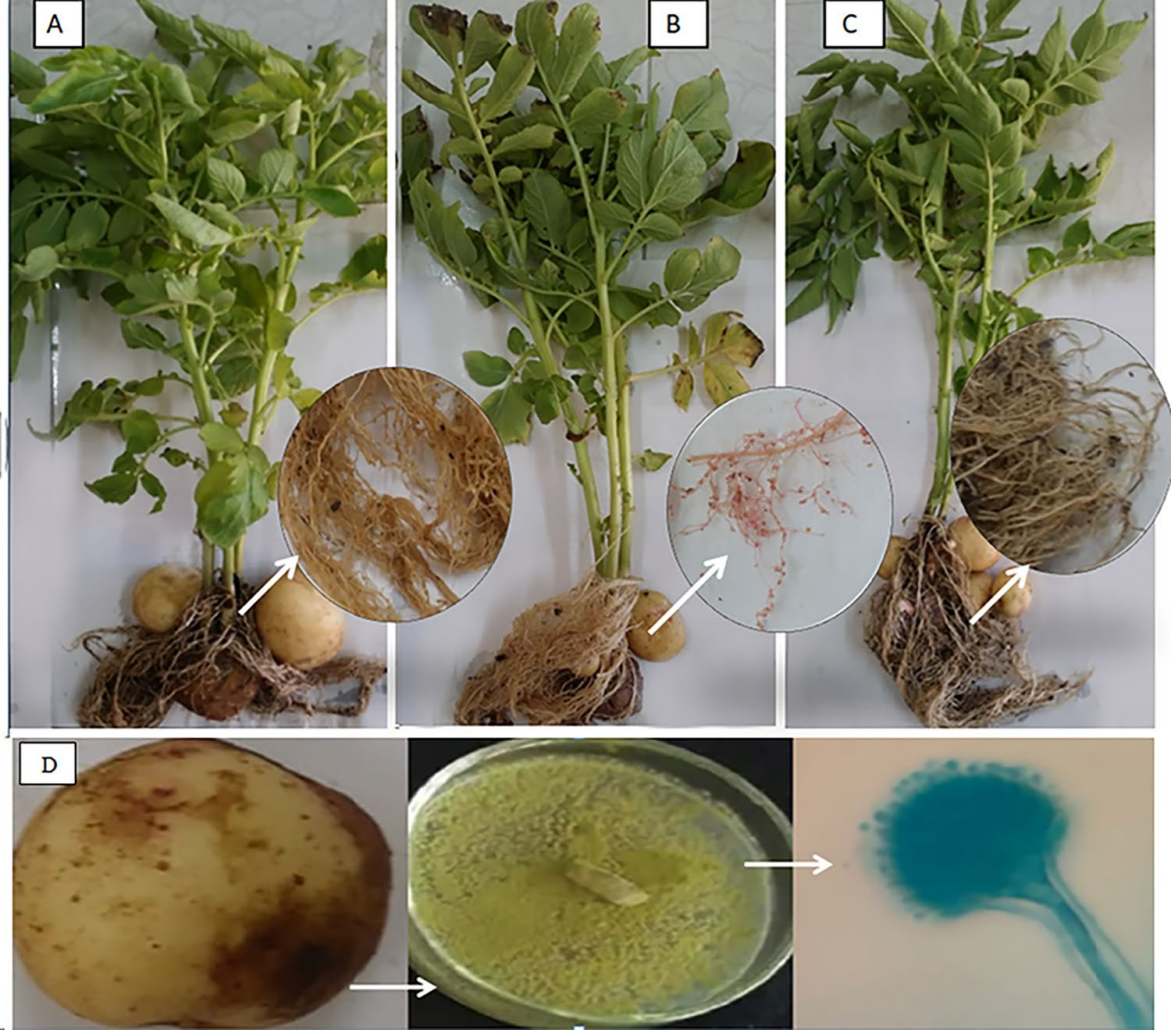
Potato plants (**A**) control healthy without any treatment, (**B**) control *M. incognita*, (**C**) treated with fungal culture filtrate at a concentration of 100% and (**D**): *A. flavus* isolated potato.

The findings in Fig. 3 indicated that the application of filtered S, S/2 (100, 75%), and abamectin concentrations increased the length and fresh weight of the plant, as well as the shoots and roots, compared to the infected control. However, there was no significant difference in shoot and root fresh weights and lengths between those treated with nematicide abamectin. The results demonstrated a noteworthy enhancement in potato growth parameters compared to the control treatment. Furthermore, potato plants treated with fungal culture filtrate at a concentration of 100% exhibited the highest percentage increase in shoot length at 41.7% Fig. 4. In comparison, those inoculated with *M. incognita* and treated with fungal filtering showed an increase of 31%.

Data in Table 1 illustrated a significant improvement (P ≤ 0.05) in potato plant growth parameters compared to the control treatment. After being inoculated with *M. incognita*, potato plants treated with *A. flavus* culture filtrate at concentrations of 50% and 100% displayed the highest percentage increase in shoot length at 20.62% and 37.85%, respectively. In comparison, Abamectin recorded an increase of only 30.9%. Additionally, potato plants treated with *A. flavus* culture filtrate at 100% and 50% concentrations showed the highest increase in root length percentages of 48.2% and 43.1%, respectively, followed by potato plants inoculated with *M. incognita* and treated with Abamectin at an increase of 43.6%. Furthermore, the highest increase in root fresh and dry weights was observed with treatments of fungal culture filtrate at a percentage increase of 15.81% and 31.41%, respectively, followed by potato plants inoculated with *M. incognita* alone and treated with Abamectin at an increasing value of 16.22%. In addition, the highest shoot dry weight increase percentages of 23.5 and 87.5% were recorded with potato plants inoculated with *M. incognita* and treated with *Aspergillus* culture filtrated at 50 and 100% concentration compare with Abamectin at 48.5% increase, respectively. Otherwise, the most significant increase percentages of 26.0% were recorded with treatments of potato plants inoculated with100% concentration from fungal filtration.

### The activity of defence-related enzymes

**Figure 5:**
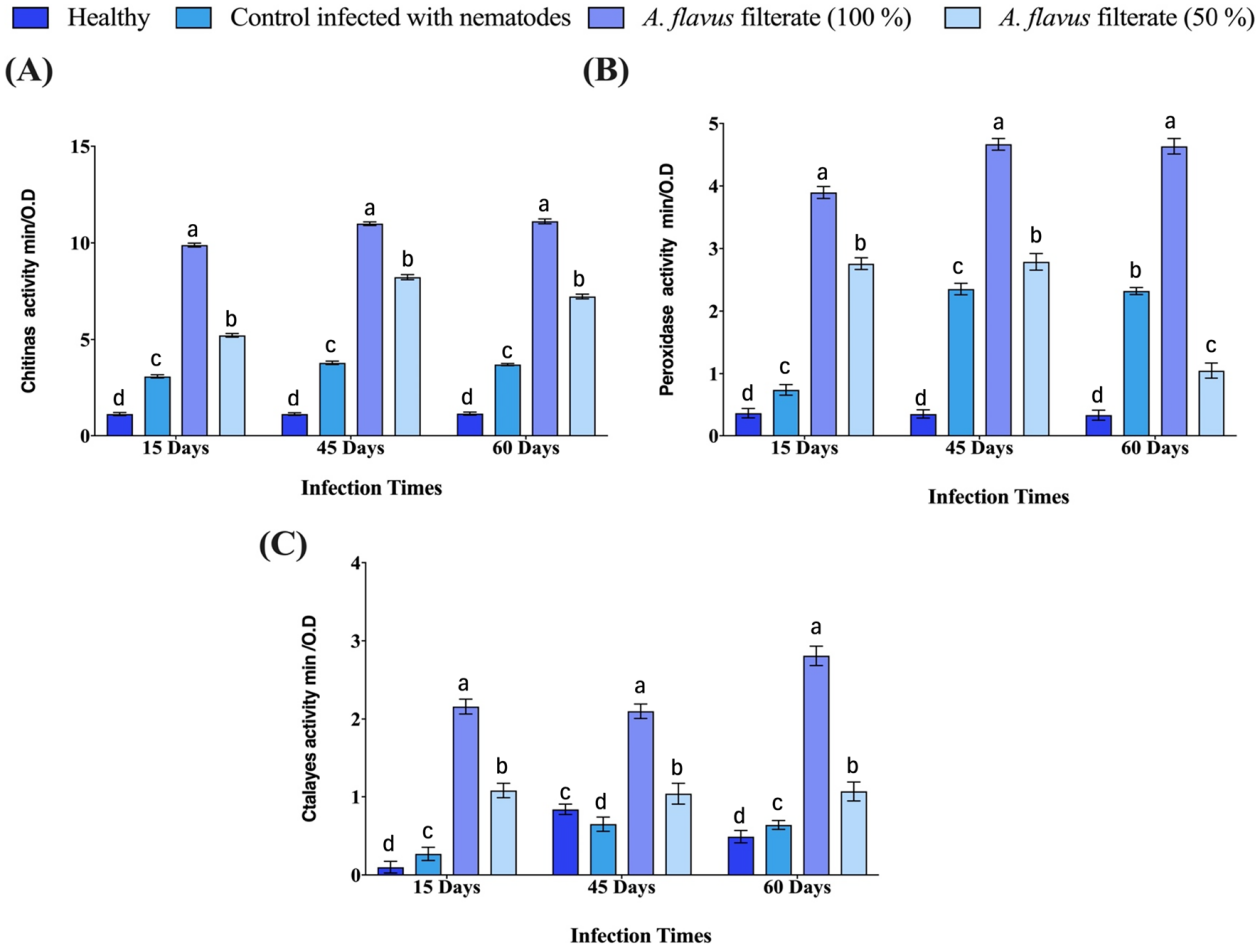
Efficacy of *A. flavus* culture filtrate with concentrations (50 and 100%) on (**A**) Chitinas extracted, (**B**) Peroxidase enzyme and (**C**) Catalyse enzyme, from the fresh potato plant inoculated with *M. incognita* under green-house conditions. Bar charts are generated using data from five biological replications with two technical replicates per replication. Each bar represents mean and error bar indicates standard deviation (± SE). This figure was drawn with GraphPad Prism 8. The significant of the changes were assessed using SAS one-way ANOVA test. Means with different letters are significantly different (P ≦ 0.05).

This study aimed to determine the relative expression levels of three defence enzymes, Chitinase, Peroxidase, and Catalase, in potato plants that were inoculated with *M. incognita* and treated with varying concentrations of *A. flavus* culture filtrate. The findings presented in Fig. 5a–c indicated that the inoculation with *M. incognit*a significantly improved the performance of Chitinase, Peroxidase, and Catalase enzymes compared to control plants. However, the two concentrations of *A. flavus* culture filtrate showed a more significant boost in defence enzyme activity than plants inoculated with *M. incognita* alone. The best treatment was observed when plants were treated with 50 mL of *A. flavus* one week before *M. incognita* inoculation as it significantly enhanced the activity of Chitinase, Peroxidase, and Catalase after 15, 45, and 60 days (9.89, 11, and 11.121), (3.89, 4.66 and 4.65), and (2.16, 2.1 and 2.81) respectively over plants inoculated with Meloidogyne only. Similarly, when *A. flavus* was mixed at a concentration of 50% with Chitinase, Peroxidase, and Catalase enzymes after meloidogyne inoculation; it resulted in increased enzyme activity after 15, 45, and 60 days (5.2, 8 0.23, and 7 0.23); (2 0.76, 2 0.79, and 1 0.05), and (1 0.08, 1 0.04, and 1 0.07) respectively over plants inoculated with *Meloidogyne* only.

### GC–MS analysis

**Figure 6:**
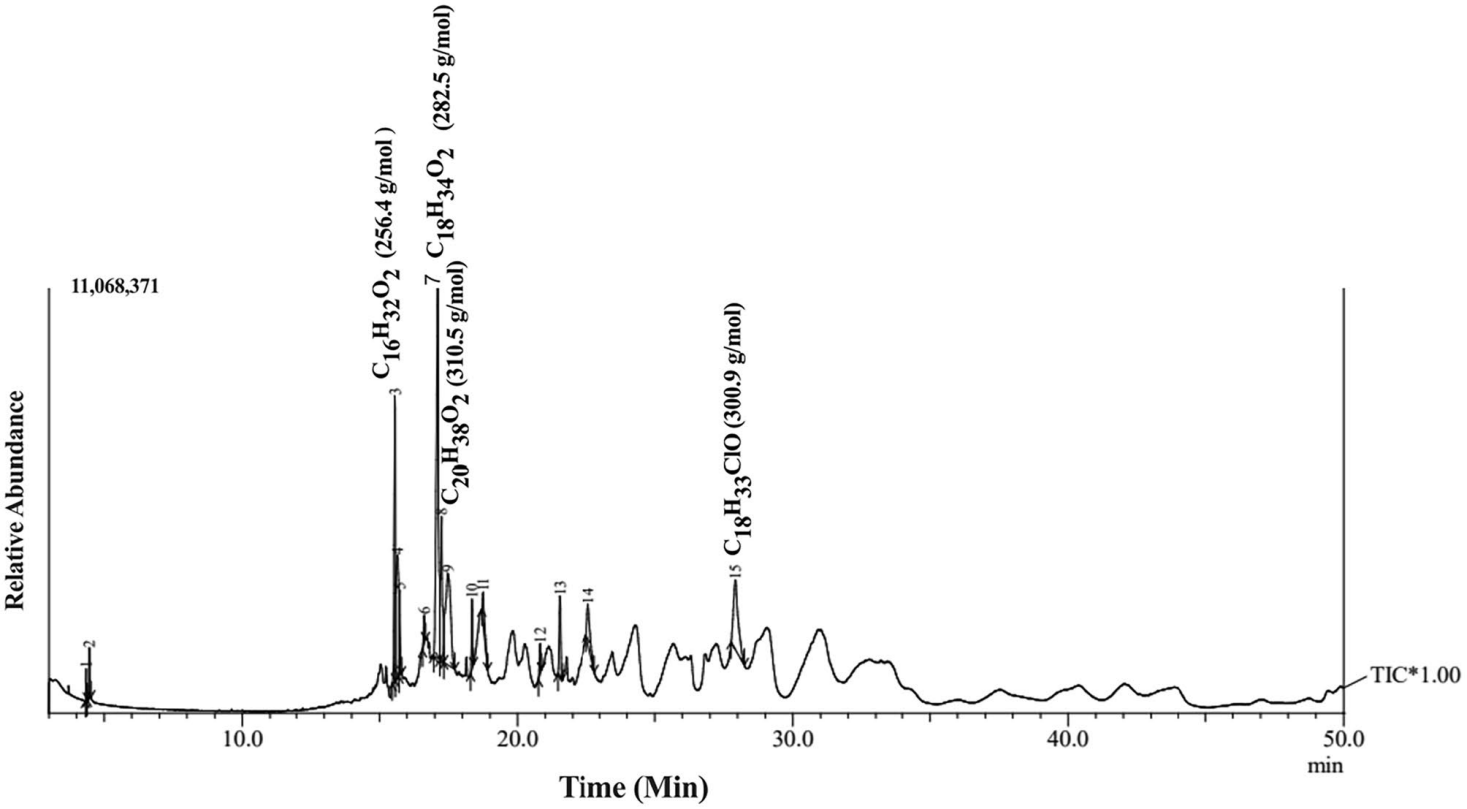
GC–Ms analysis of* A. flavus* culture filtrated using an Agilent 7693 series GC system equipped with an OV-5 capillary column and an Agilent 5975C network selective mass detector.

Capillary columns are utilized in GC–MS to separate complex mixtures containing low polarity or volatile substances. The increased fragmentation provided by GC/MS enables the identification of unknowns by searching a mass spectral database. GC–MS analysis was employed to uncover its compounds because the culture filters of the *A. flavus* fraction had low polarity. Using the NIST database with R match values exceeding 90% (Fig. 6), fifteen components were identified from the culture filter fraction of *A. flavus*. The area normalization method revealed that Gadoleic acid, Oleic acid di-ethanolamide, Oleic acid, and Palmitic acid with 310.5, 301, 282.5, and 256.4 g/mol, respectively.,accounted for 88% of the total content of the culture filter fraction from *A. flavus.*

### HPLC analysis

**Figure 7:**
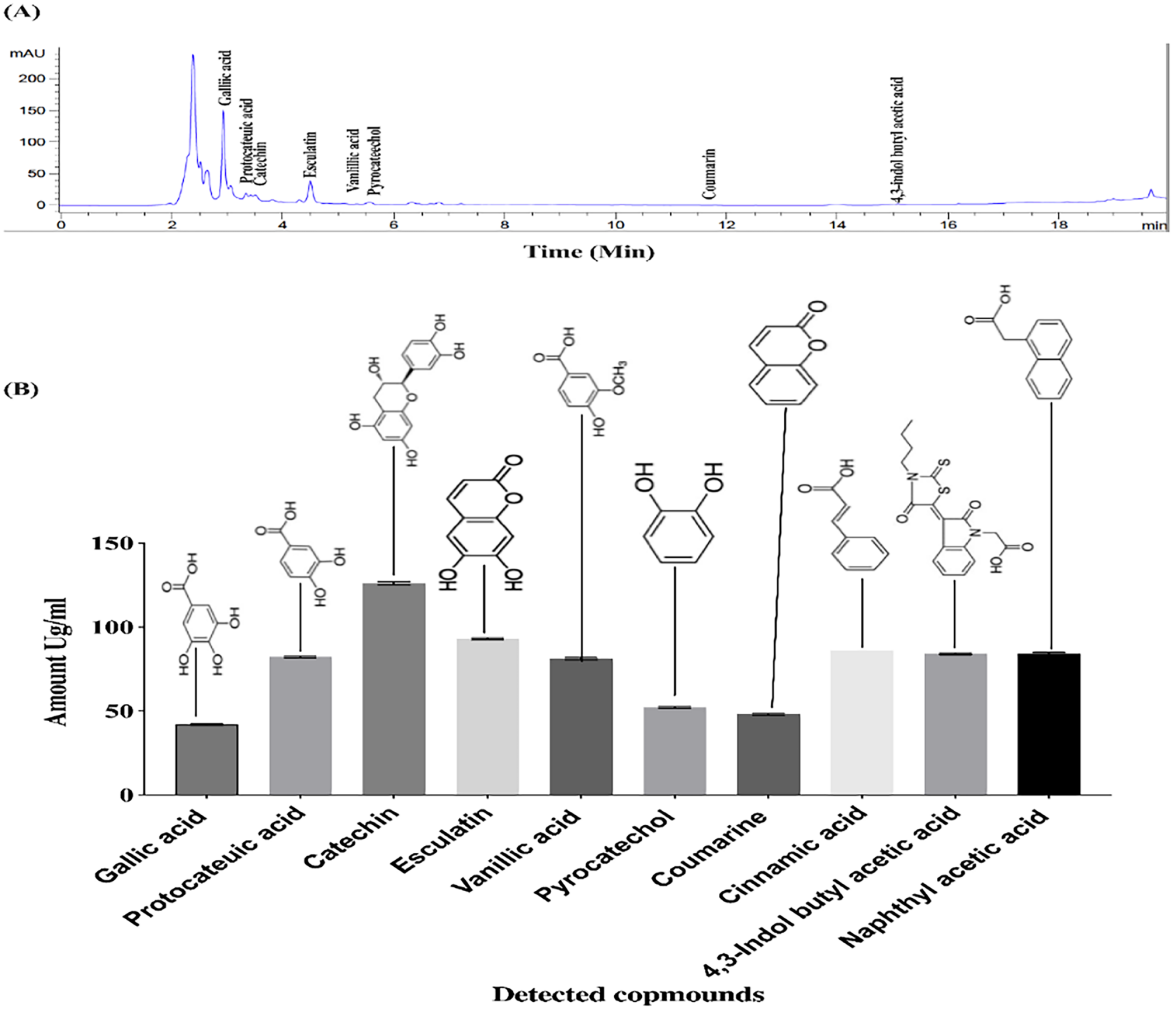
(**A**) HPLC chromatogram of *A. flavus* cultural filtrate the mobile phase consisted of a mixture of acetonitrile and 0.3% formic acid (15:85 v/v). The flow rate was set at 0.4 ml/min, and detection was performed at a wavelength of 350 nm. (**B**) The concentrations of the detected compounds. This figure was drawn with GraphPad Prism 8.

The HPLC method aimed to initially verify the presence of common phenolic antioxidants. The chromatograms indicated that the fungal extract generated several peaks that corresponded to the retention time (RT) of genuine polyphenol antioxidants. Specifically, the chromatogram for the cultural filtrate fraction from *A. flavus* strain ON146363 exhibited ten peaks that matched the RTs of authentic gallic acid (2.999 min) with a quantity of 42.000 Ug/ml, catechin (3.590 min) with 126 Ug/ml, Protocatechuic acid (3.411) with 82 Ug/ml, Esculatin (4.652 min) with 93 Ug/ml, Vanillic acid (4.979 min) with 81Ug/ml, Pyrocatechol (5.336 min) with 52 Ug/ml, Coumarine (11.613 min) with 48 Ug/ml, Cinnamic acid (13.417 min) with 86 Ug/ml, 4,3-indol butyl acetic acid (14.750 min) with 84 Ug/ml and Naphthyl acetic acid (15.697 min)with 84 Ug/ml as illustrated in Fig. 7a,b.

## Discussion

In this investigation, the culture filtrate of *A. flavus* revealed that nematicidal activity increased the mortality of J2s and decreased the egg hatchability of *M. incognita*. However, the specific percentage decrease observed in this study may be influenced by factors such as the concentration of the culture filtrate used and the specific nematode species tested. The finding that only the 10% dilution showed no significant difference compared to the water control suggests that lower concentrations of *A. flavus* culture filtrate may not have a significant impact on nematode egg hatching. This result is in agreement with what comes by^62^ who reported that the lower concentrations of fungal culture filtrates may have less pronounced effects on nematodes . The observation that juvenile mortality was highest at 100% concentration and decreased with decreasing concentrations is also consistent with previous research^62^ . This suggests that higher concentrations of *A. flavus* culture filtrate may be more effective at reducing nematode populations in lab experiment as reported by^63^.

The results of the tested fungal culture filtrate at 100% concentration in suppressing the formation of galls, egg masses, and J2s in potato plants infected with *M. incognita* are consistent with many studies that have reported the effectiveness of fungal metabolites in controlling plant parasitic nematodes. For instance, a study by^64,65^ reported that the culture filtrate of *Trichoderma harzianum* significantly reduced the galls and egg masses of *M. incognita* on tomato plants. Similarly, a study by^66^ found that the culture filtrate of *A. niger* significantly reduced the number of J2s and galls on tomato plants infected with *M. incognita.*

The effectiveness of fungal metabolites in controlling plant parasitic nematodes has been attributed to their ability to produce secondary metabolites such as enzymes, antibiotics, and volatile organic compounds that can directly or indirectly inhibit nematode growth and development^67,68^. This study's results suggest that applying filtered S, S/2 (100, 75%), and abamectin concentrations can improve the growth of potato plants infected with *M. incognita*. This is consistent with previous studies that have shown the effectiveness of these treatments in controlling nematode infestations and promoting plant growth^69,70^. The findings regarding using *A. flavus* culture filtrate are also interesting, as they suggest that this treatment can significantly increase shoot length in infected potato plants. This is consistent with previous studies that have shown the potential of fungal culture filtrates as biocontrol agents against plant pathogens^71,72^. Data from GC–MS makes it clear that *A. flavus* produces a variety of secondary metabolites with nematicidal activity that may detect it. High nematicidal activity against *M. incognita* is found within the four fatty acids identified: gadolic acid, oleic acid diethanolamide, oleic acid, and palmitic acid. The nematicidal activity of these fatty acids has been reported in many studies. A study conducted by^73^ found that gadoleic acid effectively reduced the population of *Meloidogyne incognita*, a common root-knot nematode, in tomato plants. Additionally, oleic acid di-ethanolamide, a derivative of oleic acid, has been shown to exhibit strong nematicidal activity against various nematode species. Whereas^73^ demonstrated its effectiveness against the soybean cyst nematode *Heterodera glycines.* Palmitic acid, a saturated fatty acid, was also found to have significant nematicidal effects on the root-knot nematode *Meloidogyne javanica* in a study by^74^, in this regard, various modes of action have been discussed, including overall disruption of the cell membrane permeability leading to electrolyte leakage, cell death and inhibition of protein synthesis and fatty acids metabolism^75^. Together, the combination of these substances gave rise to *A. flavus* more aggressive behavior towards nematodes. The HPLC analysis results indicated an increase in the concentration of important compounds in the *A. flavus* filtrate. This finding aligns with previous studies by^76^, who demonstrated the strong nematicidal activity of Gallic acid extracted from *Rubus niveus* against *M. incognita*, as well as the nematicidal activity of kojic acid isolated from Aspergillus oryzae^77^ and oxalic acid produced by *Aspergillus niger* F22^78^ against M. incognita. The culture filtrate of *A. flavus* JCK-4087 also exhibited potent nematicidal activity against *M. incognita*, consistent with findings by^63^. Compared to another *A. flavus* strain studied by^63^, JCK-4087 showed significantly stronger nematicidal activity with 100% J2s mortality at a concentration of 20%, whereas the other strain achieved only about 40% mortality at a concentration of 25%. The use of chemical nematicides has been effective in controlling RKNs, but prolonged use of carbamate and organophosphorus nematicides has led to nematode resistance and reduced field efficacy, prompting the development of novel chemical nematicides with different action mechanisms^79^.

The study observed an increase in the expression of defense-related genes POD, CHI, and CAT in potato plants colonized by *A. flavus*. CHI is responsible for breaking down chitin, a key component of fungal cell walls^80^. The overexpression of the antioxidant stress marker gene POD helps to reduce reactive oxygen species (ROS) in plant tissues under various stresses. This defense response was supported by the higher activities of POD and PPO enzymes. Up-regulation of these genes helped to reduce disease severity and incidence in potato plants colonized by *A. flavus*. Additionally, the accumulation of nematotoxic phenolic compounds in potato plants may hinder the growth and spread of *M. incognita* between cell^81^.

Overall, when a plant is colonized by endophytic fungi, it triggers the production of defensive secondary metabolites that help defend against pathogens. These mechanisms include cell wall lignification, production of nematicidal substances, stimulation of phytoalexins and PR-proteins, and increased expression of defense-related genes. The fungal endophyte also produces indole acetic acid (IAA) in significant amounts. Utilizing beneficial endophytic microorganisms as bio-fertilizers can enhance plant growth and production for sustainable agriculture and food safety^81^. These results are consistent with previous studies that have examined the impact of plant–microbe interactions on defense enzyme activity such as^82^. For instance^34,83^, research demonstrated that introducing the beneficial fungus *Trichoderma harzianum* increased Peroxidase and Catalase activity in tomato plants. At the same time^84^ studies found that introducing the beneficial bacterium *B. subtilis* increased Chitinase and Peroxidase activity in some plants. The enzyme Catalase breaks down hydrogen peroxide into water and oxygen, as noted by^85^. It has an incredibly high turnover rate, with one molecule of Catalase able to convert 40 million molecules of hydrogen peroxide into water and oxygen every second, according to^86^. Peroxidases are involved in several physiological processes, including auxin catabolism, defense against pathogens, respiratory processes, and the synthesis of lignin and other terpenoids that contribute to phytoalexin production^87^. The activity of oxidative enzymes has been tested with nematode infection and has proven useful as a biochemical marker in various plant species, such as tomato, as demonstrated by^88^.

## Conclusion

In summary, the findings suggest that the *A. flavus* (ON146363) cultural filtrate may positively boost the activity of defensive enzymes and improve a plant's ability to resist plant pathogenic nematodes. Fungal filtrate is also rich in components that prevent or effectively control nematodes. This makes it a promising solution for controlling nematodes, particularly in severe cases. Additionally, it is eco-friendly and serves as a bio-agent for the ecosystem. In conclusion, the tested fungal culture filtrate suppresses *M. incognita* infestation in potato plants, consistent with many studies that have reported the potential of fungal metabolites as an alternative to synthetic nematicides.

## Data Availability

All data generated or analyzed during this study are included in this article.
